# A Comparison of Health Risks from PM_2.5_ and Heavy Metal Exposure in Industrial Complexes in Dangjin and Yeosu·Gwangyang

**DOI:** 10.3390/toxics12020158

**Published:** 2024-02-18

**Authors:** Jeong-In Jeon, Ji-Yun Jung, Shin-Young Park, Hye-Won Lee, Jeong-Il Lee, Cheol-Min Lee

**Affiliations:** 1 Department of Chemical and Environmental Engineering, Seokyeong University, Seoul 02713, Republic of Korea; hhzz01@skuniv.ac.kr (J.-I.J.); jju1049@skuniv.ac.kr (J.-Y.J.); tlsdud060900@skuniv.ac.kr (S.-Y.P.); 2 Institute of Environment and Health, Seoul 02713, Republic of Korea; gpdnsis@naver.com; 3 Department of Nano, Chemical and Biological Engineering, Seokyeong University, Seoul 02713, Republic of Korea; emjilee@skuniv.ac.kr

**Keywords:** PM_2.5_, heavy metals, health risk assessment, industrial complexes, Republic of Korea

## Abstract

Particulate matter (PM) can cause illness, including respiratory diseases, and PM_2.5_ compositions are likely to vary according to the emission profiles of industrial complexes. This study analyzed and compared the concentrations and distributions of PM_2.5_ and heavy metals in two regions of Republic of Korea: Yeosu·Gwangyang, which houses a massive national industrial complex, and Dangjin, which houses power plants. Further, we conducted a health risk assessment on the residents of the areas near these industrial complexes. Measurements were taken at five different points in each setting over a two-year period from August 2020 to August 2022. We found differences in PM_2.5_ concentrations and heavy metal composition ratios across the sites. Specifically, PM_2.5_ concentrations exceeded the standard of 1 at all measurement sites, while the specific heavy metals exceeding the standard varied across the sites. Ultimately, we observed regional differences in PM_2.5_ composition across measurement sites across and within the two regions and variations in health risks and according health effects due to the absence of PM_2.5_ toxicity values, and compared the health risks of two industrial complexes with different characteristics. These findings underscore the importance of considering not only PM_2.5_ but also its composition in exposure and health risk assessments.

## 1. Introduction

Particulate matter (PM), a crucial indicator for assessing atmospheric air quality, refers to particles that exist in the atmosphere in a solid or liquid form [1,2]. PM is primarily composed of ions and contains various chemical substances, including organic components and trace metals [3,4]. It is well established that PM in the atmosphere has varying adverse impacts on the human body depending on the composition and substances of the particulates [5,6,7,8]. For example, PM_2.5_ is much smaller than PM_10_, and has been reported as penetrating deep into the respiratory system, reaching the alveoli of the lungs and leading to conditions such as heart diseases and respiratory disorders [9]. In 2013, the World Health Organization (WHO) classified PM_10_ and PM_2.5_ as Group 1 carcinogens—equivalent to substances such as asbestos and arsenic—owing to their abilities to penetrate not only the respiratory system but also the skin, and to seriously harm the human body. Since this designation, there has been continuous interest in, and research on, PM in Republic of Korea [9] and beyond.

PM_2.5_ is divided into primary particles emitted in solid form from sources such as industrial stacks, power plants, and vehicle exhausts, and secondary particles generated through chemical reactions between gaseous substances, such as sulfates and nitrates, released from sources and other substances in the air. Additionally, anthropogenic sources encompass industrial facilities and motor vehicles, while natural sources include events such as fires, yellow dust, and volcanic eruptions [9]. Therefore, it is expected that there will be differences in PM_2.5_ components depending on the characteristics of industrial complex emissions, and since there are differences in health effects depending on the composition, it is judged that research on components as well as PM_2.5_ concentration is necessary for long-term management. However, studies on the health effects of PM_2.5_ conducted in urban and industrial areas have mainly been conducted as PAHs studies regarding carcinogenicity, and studies comparing the health effects of PM_2.5_ and exposure to the middle genus in industrial complexes with different characteristics have been insufficient.

In a study investigating the bio-accessibility of PM_2.5_ and heavy metals in body fluids, Expósito et al. reported that Cu, Mn, V, and As had high bio-accessibility and were therefore likely to impact health [10]. Although the human body is resistant to heavy metals and accordingly equipped with a mechanism to eliminate them, many prior studies have reported that a high concentration of heavy metals can adversely health. In addition, prolonged exposure to low concentrations have also been reported to include renal dysfunction, chronic respiratory disorders, and skeletal and cardiovascular diseases [11,12].

Industrial complexes may exhibit distinct chemical characteristics compared to typical urban areas, primarily due to variations in fine particle emissions. Specifically, the ambient air around industrial complexes often contains substantial amounts of fine particles in addition to the gaseous pollutants these facilities emit, such as sulfur dioxide and nitrogen oxides; therefore, the air around industrial complexes can harm the environment and human health [13]. This paper reports a study on the varying concentrations, compositions, and health risk assessments of PM_2.5_ and heavy metals in different industrial regions of Republic of Korea. As of 2022, there were over 1200 industrial complexes in Republic of Korea [14]. Industrial complexes have proliferated in the nation due to their cost-effective management, government financial support, and flexible compliance. However, these complexes pose environmental and health challenges because individual point sources of pollution can aggregate within them and create substantial area sources of pollution.

Against this background, this study aimed to compare PM_2.5_ and heavy metal compositions in two different industrial complexes in Republic of Korea and conducted health risk assessments on residents in the vicinity to compare the compositions and consequent health risks according to the characteristics of the complexes.

## 2. Materials and Methods

### 2.1. Measurement Sites

**Figure 1:**
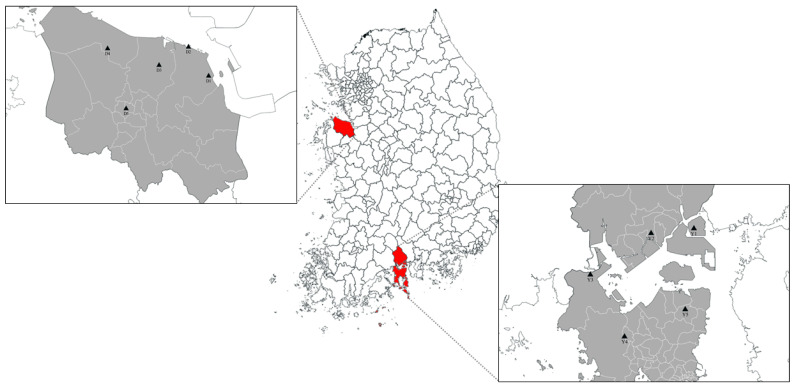
Measurement sites in this study.

We studied the regions of Yeosu·Gwangyang, which houses massive national petrochemical and steel industrial complexes, and Dangjin, which houses power plants. Measurements were taken from five sites in each region to comparatively analyze the health impact of PM_2.5_ according to the different characteristics of the complexes. Table S1 and Figure 1 describes these sites.

Yeosu and Gwangyang are industrial cities situated along the coast. They both host multiple industrial complexes with a diverse array of facilities that emit various air pollutants. As of April 2023, the petrochemical industry is the most common industry (45.4%) in Yeosu, followed by the machinery (25.3%) and non-manufacturing (17.8%) industries. In Gwangyang, non-manufacturing industry takes the lead (26.3%), followed by the machinery (23.2%) and steel (17.4%) industries [14].

Dangjin is densely populated with large-scale emission facilities, such as steel mills and power plants, which contribute to air pollution. According to a Ministry of Environment report [15], Dangjin emitted 16,238 tons of air pollutants in 2020 and 13,760 tons in 2021; these annual emission rates were the third-highest nationwide.

### 2.2. Collection and Analysis of Data on Particle Composition and Concentration

Measurements were taken over a two-year period from August 2020 to August 2022. Measurements were obtained twice a week during the winter, when PM is high, and the spring, when yellow dust strikes the country, and once a week during the summer and fall [16,17]. When samples were lost due to weather conditions, such as typhoons and heavy rainfall, or equipment malfunctions, the number of lost samples was deducted from the total samples collected, and the lost samples were excluded from the analysis.

PM_2.5_ samples were collected using a PM_2.5_ sampler (PMS-204, APM Co., Bucheon-si, Republic of Korea) at a flow rate of 16.7 L/min over a 24-h period. Teflon filters (PTFE 2.0 μm, 47 mm) were used to analyze the heavy metal composition of PM_2.5_. Filters were weighed before and after sampler collection following a 24-h constant temperature and humidity conditioning process, and mass concentrations of PM_2.5_ were corrected using blank samples. The measurements and analyses were conducted in accordance with the *Guidelines for the Installation and Operation of Air Pollution Monitoring Networks* provided by the National Institute of Environmental Research (NIER).

Nineteen trace elements were analyzed (Al, Ti, V, Mn, Fe, Ni, Co, Cu, Zn, As, Sr, Mo, Cd, Ba, Pb, P, S, Cr, Si) using the Energy Dispersive X-ray Fluorescence Analyzer (ED-XRF, Epsilon4, Malvern Panalytical, Almelo, The Netherlands). Metal concentrations were calculated as the mass of the element per cubic meter of air, adjusted to standard conditions of 0 °C and 760 mmHg. The equipment specifications of ED–EXF used in this study are shown in Table 1 below.

### 2.3. Quality Assurance and Control

To evaluate the reliability of the results, a quality assurance/quality control (QA/QC) protocol was used. The method detection limit (MDL) and relative standard deviation (RSD) were determined based on the *Guidelines for the Installation and Operation of Air Pollution Monitoring Networks*. Table S2 shows the QA/QC results. For the quality control of each substance, calibration curves were prepared at three or more concentrations using Nucleipore’s Aerosol Membrane. MDL was calculated by measuring 7 standard solutions, and RSD was calculated by measuring 4 samples to which standard substances were added (concentration 1 to 5 times the MDL). The internal quality control cycle was performed once a year.

### 2.4. Health Risk Assessment

Conventionally, health risk assessments related to PM exposure are primarily focused on components such as heavy metals and ions in PM due to a lack of PM toxicity, as opposed to analyzing the health risks of the PM itself. To address the lack of PM toxicity data, we adopted the methodology proposed by Amnuaylojaroen and Parasin (2023) [18] and set the toxicity reference value to the annual average of 15 μg/m^3^ in Republic of Korea in order to calculate the health risks from PM exposure. Furthermore, we conducted health risk assessments targeting residents of areas in the vicinity of industrial complexes and categorized the residents into three groups—children, adult men, and adult women—for comparison. More specifically, we assessed health risks from exposure to PM_2.5_ and heavy metal inhalation. Five heavy metals with available toxicity data—Mn, Ni, As, Cd, Cr^6+^—were included. Additionally, we conducted separate health risk assessments for carcinogens and non-carcinogens. Table 2 shows the toxicity data of the substances.

Risk was determined using excess cancer risk (ECR) for carcinogens and the hazard quotient (HQ) for non-carcinogens. ECR is the product of the cancer slope factor (CSF) and lifetime average daily dose (LADD); meanwhile, HQ is the quotient of the average daily dose (ADD) divided by the reference dose (RfD). We converted CSF into unit risk (UR) adjusted for body weight and inhalation rate and RfD into reference concentration (RfC) adjusted for body weight and inhalation rate. The calculations are detailed in Equations (1) and (2).

(1) $$CSF({\left(mg/kg/day\right)}^{-1})=\frac{UR({\left(\mu g/m^{3}\right)}^{-1})\times BW(kg)}{IR(m^{3}/day)}\times \frac{1000\;\mu g}{1\;mg}$$

(2) $$RfD(mg/kg/day)=\frac{RfC(mg/m^{3})\times IR(m^{3}/day)}{BW(kg)}$$

For carcinogens, lifetime exposure from carcinogenesis or chronic effects was assumed, and LADD was calculated with life expectancy using Equation (3). For non-carcinogens, ADD was calculated using Equation (4). For both LADD and ADD, we assumed that 100% of the exposed amount was absorbed by the body.

(3) $$LADD=\frac{C\times IR\times EF\times ED}{BW\times LT}$$

(4) $$ADD=\frac{C\times IR\times EF\times ED}{BW\times AT}$$

where LADD is the lifetime average dose (mg/kg/day), C is the pollutant concentration (mg/m^3^), IR is the inhalation rate (m^3^/day), EF is exposure frequency (day/year), ED is exposure duration (year), BW is body weight (kg), LT is average exposure time (day), ADD is average daily dose (mg/kg/day), and AT is average exposure time (day). Table 3 shows the exposure factors used in the exposure assessment and the data sources. The exposure factors for BW and IR among adults were derived from the Korean Exposure Factor Handbook published by NIER (2019) [19]. The exposure factors for BW and IR among children were obtained from the Institute’s Korean Children’s Exposure Factor Handbook (NIER, 2019) [20].

Risks were assessed using ECR for carcinogens. With a reference level set a 1.00 × 10^−6^ (excess cancer risk per 1,000,000 individuals), as specified by the US EPA, we situated ECR values exceeding this threshold as indicative of a potential health risk. For non-carcinogens, a threshold of 1 was used, where HQ values exceeding 1 were deemed to indicate potential risk.

(5) ECR=CSF×LADD

(6) $$HQ=\frac{ADD}{RfD}$$

## 3. Results

### 3.1. PM_2.5_ and Component Concentrations

Figure shows the PM_2.5_ and component concentrations obtained at each measurement site. In Dangjin, the mean PM_2.5_ concentration was 24.84 ± 13.71 µg/m^3^ at D1, 24.36 ± 14.17 µg/m^3^ at D2, 21.76 ± 12.37 µg/m^3^ at D3, 20.92 ± 12.67 µg/m^3^ at D4, and 21.39 ± 13.06 µg/m^3^ at D5. In Yeosu·Gwanayang, the mean PM_2.5_ concentration was 16.71 ± 8.73 µg/m^3^ at Y1, 16.21 ± 10.14 µg/m^3^ at Y2, 19.04 ± 9.79 µg/m^3^ at Y3, 17.18 ± 9.02 µg/m^3^ at Y4, and 18.13 ± 9.64 µg/m^3^ at Y5. The mean PM_2.5_ concentrations in all measurement sites in Dangjin and Yeosu·Gwangyang did not exceed the national daily average of 35 µg/m^3^ but did exceed the annual average of 15 µg/m^3^. Furthermore, PM_2.5_ concentrations were higher in Dangjin than Yeosu·Gwangyang.

**Figure 2:**
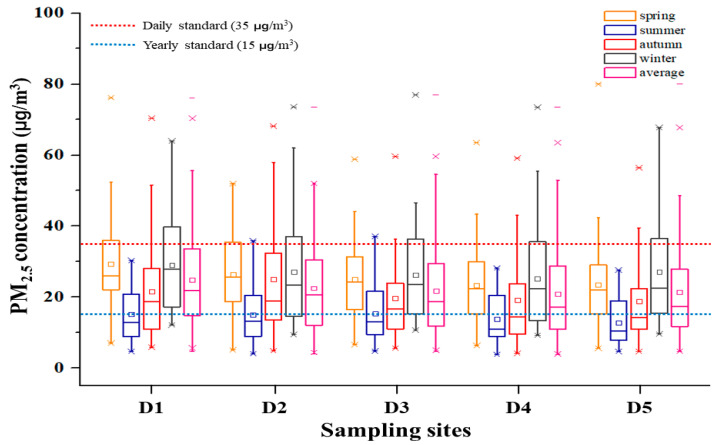

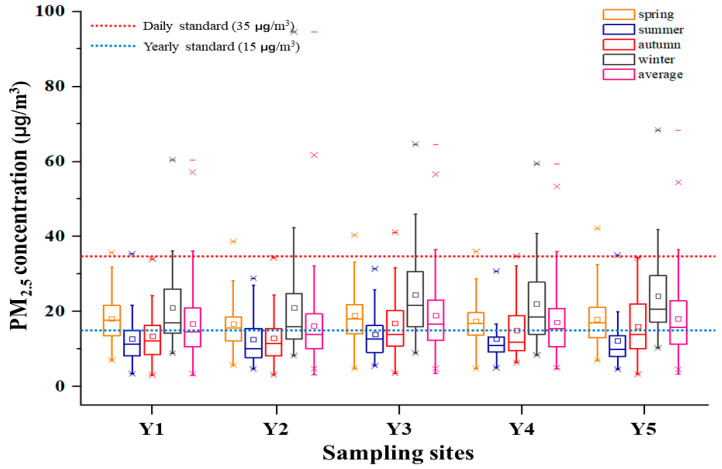
PM_2.5_ concentrations by season at each measurement site.

Figure 2 shows the PM_2.5_ concentrations by season at each site. In Dangjin, the mean concentrations in the spring, summer, fall, and winter were 25.51 ± 11.76 µg/m^3^, 14.42 ± 7.64 µg/m^3^, 20.89 ± 14.06 µg/m^3^, and 26.93 ± 14.10 µg/m^3^, respectively. In Yeosu·Gwangyang, the mean concentrations were 17.88 ± 6.79 µg/m^3^, 12.85 ± 6.72 µg/m^3^, 14.93 ± 7.72 µg/m^3^, and 22.58 ± 12.10 µg/m^3^, respectively. At all 10 measurement sites, the concentration was the highest in the winter, second-highest in the spring, third-highest in the fall, and lowest in the summer; these findings are consistent with those of Hwang et al. (2021) [16], who found that PM concentrations are highest in the winter (due to heating combustion) and spring (due to yellow dust).

At all sites, PM_2.5_ concentrations were highest in the winter and spring. This is attributable to the fact that Republic of Korea is heavily influenced by the inland areas of China during the winter and spring due to the prevailing westerly winds; in particular, the yellow dust storms that occur in the spring contribute to the high concentrations [22]. These results are similar to those of Cho et al. (2016) [23], who reported that PM_2.5_ concentrations were high in the winter and spring in Chuncheon and Yeongwol; the findings of Hwang et al. (2019) [24], who measured PM_2.5_ concentrations in industrial complexes in Gyeongbuk; and the findings of Kim et al. (2023) [25], who found that PM_2.5_ concentrations in the Ansan and Siheung industrial complexes were high in the winter and spring.

To determine whether there are significant differences in heavy metal concentrations at each measurement site between Dangjin and Yeosu·Gwangyang, we performed a one-way analysis of variance (ANOVA), followed by a Scheffe post-hoc test to identify specific sites with different concentrations.

In Dangjin, the concentrations of V, Mn, Fe, Ni, Co, Cu, Zn, Pb, and Cr significantly differed across measurement sites (*p* < 0.05). The post-hoc test revealed that the concentration of V was significantly different at D2 compared to all other sites and that the concentration of Mn was significantly different at D1 compared to all other sites. V is generated from the combustion of petroleum fuel [26], while Mn is released from steel and non-ferrous metal-related facilities [27]. Hence, our results may be attributed to the fact that Asan national industrial complex, which houses many steel and petrochemical plants, and the Godae·Bugok districts are in the vicinities of D1 and D2.

In Yeosu·Gwangyang, the concentrations of V, Mn, Fe, Ni, Co, Cu, Zn, As, Mo, P, S, and Cr significantly differed across the measurement sites (*p* < 0.05). The post-hoc test revealed that the concentration of Mn was significantly different at Y2 compared to all other sites, and that the concentrations of Fe, Ni, Co, As, and Cr were significantly different at Y1 compared to all other sites. In addition, the concentration of P was significantly different at Y5 compared to all other sites. These results may be due to several reasons. Manshaet et al. [28] and Singh et al. [29] reported that Fe, Ni, Co, As, and Cr are emitted by the iron and steel industries, and the Gwangyang steelmaking complex is located in the vicinity of Y1. Additionally, Singh et al. [29] reported that Mn is emitted by automotive fuel additives, and the Yulchon industrial complex—which houses many automobile and trailer manufacturers—is located near Y3.

The heavy metal components of PM_2.5_ were compared across measurement sites between Dangjin and Yeosu·Gwangyang, and there were differences in the specific compositions of PM_2.5_ according to the characteristics of each measurement site. As reported by Li et al. [30], this suggests that PM_2.5_ compositions are influenced by regional characteristics. Therefore, these findings highlight the need to consider regional characteristics—specifically, regional hazardous chemical component data—when evaluating the health impacts of PM_2.5_ exposure.

**Figure 3:**
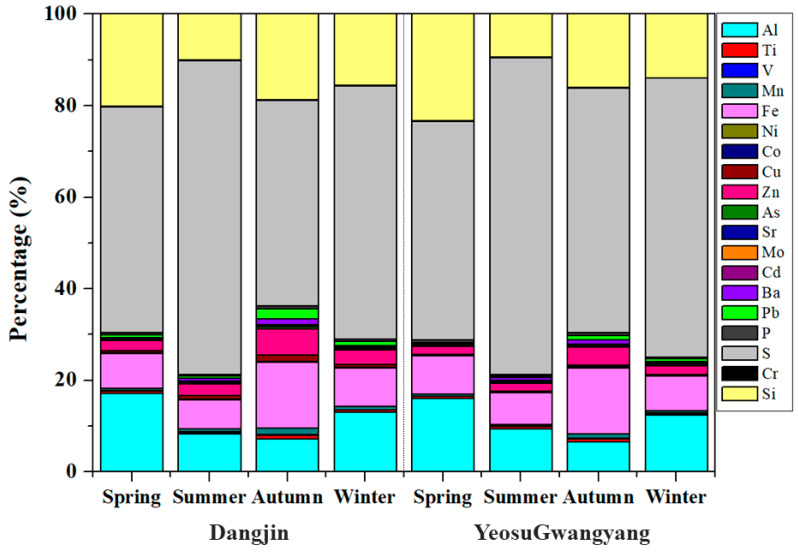
Pollutant proportions by season in Dangjin and Yeosu·Gwangyang.

Figure 3 shows the percentages of each heavy metal component by season. The highest proportions of crustal elements, such as Al and Si, were found during the spring; this result is likely to be due to the influence of dry atmospheric conditions and yellow dust originating from deserts or plateaus [26]. The highest proportions of components known to have anthropogenic origins, such as Ni, Zn, and Pb, were found in the fall and winter, consistent with the finding of Jo et al. [31].

### 3.2. Health Risk Assessment for Non-Carcinogens

**Figure 4:**
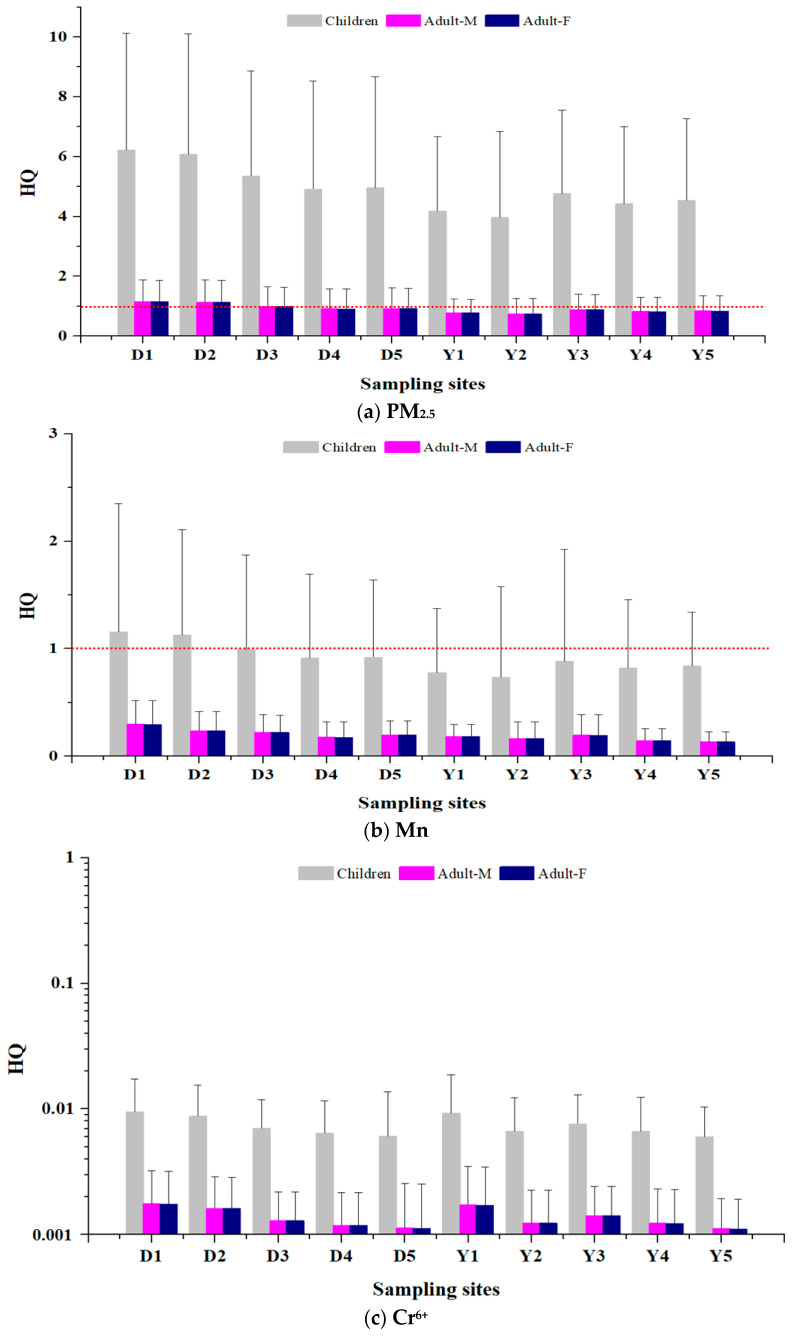
Non-carcinogenic risk by measurement site. Dashed red line: Thresholds for assessing health effects of non-carcinogenic.

HQ was calculated for the non-carcinogens selected for the health risk assessment in this study (PM_2.5_, Mn Cr^6+^) (Table S5, Figure 4). PM_2.5_ exceeded the threshold of 1 at all measurement sites in all study groups (children, adult men, adult women), suggesting a potential health impact. In addition, HQ was higher in children than adults at all measurement sites. In Dangjin, children’s HQ was the highest at D1 (6.22) and the lowest in D4 (4.91). In Yeosu·Gwangyang, children’s HQ was the highest at Y3 (4.76) and lowest at Y2 (3.96).

The HQ for Mn did not exceed the threshold of 1 in adults at all measurement sites in Dangjin and Yeosu·Gwangyang, suggesting no potential health impact. However, the HQ for Mn in children exceeded the threshold of 1 at D1, D2, D3, D5, and Y3, with 1.60, 1.27, 1.20, 1.06, and 1.06, respectively.

The HQ for Cr^6+^ did not exceed the threshold of 1 in both children and adults at all measurement sites in Dangjin and Yeosu·Gwangyang, suggesting no potential health impact.

### 3.3. Health Risk Assessment for Carcinogens

**Figure 5:**
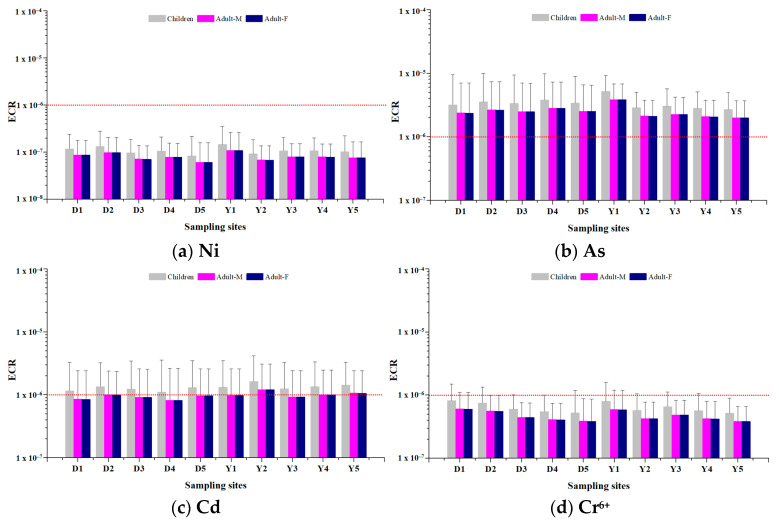
Carcinogenic risk by measurement site. Dashed red line: Thresholds for assessing health effects of carcinogenic.

ECR was calculated for the carcinogens selected for health risk assessment in this study (Ni, As, Cd, Cr^6+^) (Table S6, Figure 5).

The ECR for Ni did not exceed the threshold of 1.00 × 10^−6^ in all study groups (children, adult men, adult women) at all measurement sites in Dangjin and Yeosu·Gwangyang. Furthermore, ECR in children was higher than that in adults at all measurement sites.

The ECR for As exceeded the threshold of 1.00 × 10^−6^ in all study groups at all measurement sites in Dangjin and Yeosu·Gwangyang but did not exceed the permissible risk threshold of 1.00 × 10^−4^ proposed by the US EPA.

The ECR for Cd exceeded the threshold of 1.00 × 10^−6^ in children at all measurement sites in Dangjin and Yeasu·Gwangyang. In adult men, it exceeded the threshold of 1.00 × 10^−6^ at D2, Y2, Y4, and Y5 (1.00 × 10^−6^, 1.21 × 10^−6^, 1.00 × 10^−6^, 1.06 × 10^−6^, respectively).

The ECR for Cr^6+^ did not exceed the threshold of 1.00 × 10^−6^ in all measurement sites in Dangjin and Yeosu·Gwangyang. Furthermore, ECR in children was higher than that in adults at all measurement sites.

## 4. Discussion

The mean PM_2.5_ concentration was higher in Dangjin (22.62 ± 13.24 µg/m^3^) than Yeosu·Gwangyang (17.45 ± 9.49 µg/m^3^). This difference can be attributed to the increased air pollution due to the presence of several major emission facilities in the Chungnam region, where Dangjin is located, including coal fired power plants, Hyundai Steel, and the Daejeon Petrochemical Complex, and to the dispersion of coal produced from processes at the Dangjin Steel Mill (e.g., blast furnaces, steel making, sintering, and pelletizing) to nearby areas [32]. Furthermore, owing to the geographical advantage of Dangjin, which is connected to inland steel mills, industrial complexes, power plants, and roads, sources of pollution are concentrated in this limited area. Additionally, as suggested by Son et al. (2020) and Kim et al. (2020), these results may be due to the presence of the Charyeong Mountains, which may prevent the dispersion and dilution of pollutants into downwind areas with the prevailing westerly or northwesterly winds and, instead, causes the pollutants to be localized to the area [33,34].

We performed Student’s *t*-tests to examine the differences in PM_2.5_ concentrations between Dangjin and Yeosu·Gwangyang. The findings revealed significant differences in the PM_2.5_ concentrations between the two regions (*p* < 0.05). The differences in these concentrations appear to be due to the differences in the major industries between the Dangjin and Yeosu·Gwangyang industrial complexes. We also analyzed the differences in the heavy metal compositions of PM_2.5_ between the two regions. We uncovered significant differences in the concentrations of Mn, Cu, Zn, As, Mo, and Pb between the two regions (*p* < 0.05). The concentrations of Mn, Cu, Zn, As, and Pb were higher in Dangjin than in Yeosu·Gwangyang. In particular, the concentration of Cu was 4.3-fold higher in Dangjin than in Yeosu·Gwangyang. In Dangjin, Cu was highest in D4; D4 is near the Seokmun national industrial complex, which houses many steel and machinery plants that could have contributed to the results [14]. Meanwhile, Mn, Cu, and As have been reported to be generated by coal combustion [27]. Particularly, As is a major metabolite of bituminous coal combustion, and As, Cu, and Fe have also been reported to be released from iron smelting furnaces [35]. Hence, the high concentration might be influenced by the presence of the Dangjin thermal power plant [32,33].

In Yeosu·Gwangyang, the proportion of Fe was higher at Y1 (12.6%) than in the other sites. Fe is a crustal element and is emitted by various sources in industrial complexes. Kang et al. (2018) [36] and Choi et al. (2023) [37] reported that Fe concentrations were consistently higher in steelmaking industrial complexes, echoing our results. The Gwangyang national industrial complex is located near Y1, and the fact that the Gwangyang national industrial complex is the largest steelmaking industrial complex in South Jeolla Province with a designated area of 96,405,000 m^2^ [27] might have contributed to the high F2 proportion in Y1.

We observed differences in PM_2.5_ and heavy metals with potential health risks at different measurement sites. Mn exceeded the threshold of 1 in children at D1, D2, D3, D5, and Y3. Cd exceeded the threshold of 1.00 × 10^−6^ in adult mean at D3, Y2, Y4, and Y5 and in adult women at D2, Y2, and Y5. Cd exceed the threshold of 1.00 × 10^−6^ in children in all measurement sites, and As exceeded the threshold of 1.00 × 10^−6^ in children, adult men, and adult women in all measurement sites. Exposure to elevated levels of inorganic As is associated with irritation of the skin and mucous membranes. Although Mn was nutritionally essential in humans at low level, bad effects may be caused on the nervous system when people are exposed to high level [38]. Cd, when inhaled, accumulates in the liver and kidneys through the lungs while in the atmosphere. Cd is carcinogenic and is known to have harmful effects on respiratory diseases, high blood pressure, the nervous system, and tubular functions of the liver and kidneys [39].

In our carcinogen health risk assessment, ECR was highest for As, second-highest for Cd, third-highest for Cr^6+^, and lowest for Ni. Meanwhile, Li et al. (2022) [30] reported that ECR was highest for As, second-highest for Cr^6+^, third-highest for Cd, and lowest for Ni in adult men, adult women, and children. Morakinyo et al. (2021) [40] measured PM_2.5_ heavy metal concentrations in Gauteng industrial complexes in South Africa and conducted health risk assessments; they found that Cr posed the highest health risk, As the second-highest, Cd the third-highest, Ni the fourth-highest, Pb the lowest. The current study found that PM_2.5_ component concentrations vary across regions and different sites within the same region, suggesting that health risks may also differ across regions due to the variations in the compositions. Thus, PM_2.5_ compositions and concentrations should be considered when conducting exposure and health risk assessments.

This study had some limitations. Although the exposure coefficients used for PM_2.5_ and heavy metal health risk assessments, such as body weight, inhalation rate, exposure duration, and average lifespan, may differ across regions and individuals, it is notable that our findings were based on the average values presented in the *Korean Children’s Exposure Factor Handbook* and *Korean Exposure Factor Handbook*, which might have resulted in over- or under-estimation. Furthermore, we analyzed 19 heavy metals but only included five with available toxicity data (Mn, Ni, As, Cd, Cr^6+^) in the health risk assessment. Hence, the results may not fully reflect the actual health impact of the metals on residents near the industrial complexes. Additionally, due to the lack of toxicity data for PM_2.5_, we set the reference value to 15 µg/m^3^, the annual average in Korea, for our health risk assessment—this can add uncertainty to the results. However, despite these limitations, our findings can still serve as foundational data for developing air quality control policies in Korea. Additionally, the findings may be used as baseline data for conducting health risk assessment for PM_2.5_ concentrations and components that consider the diverse characteristics of industrial complexes.

## 5. Conclusions

This study aimed to shed light on the need for health risk assessments for PM_2.5_ and its components by considering the characteristics of different industrial complexes in Republic of Korea. Specifically, we investigated PM_2.5_ and its compositions in Dangjin and Yeosu·Gwangyang, where there are industrial complexes with varying characteristics. We also conducted health risk assessments related to PM_2.5_ exposure as well as its components among residents of areas in the vicinity of these industrial complexes and compared the health risk.

The mean PM_2.5_ concentrations in Dangjin and Yeosu·Gwangyang were 22.65 ± 13.24 µg/m^3^ and 17.45 ± 9.49 µg/m^3^, respectively; the mean concentration significantly differed between the two regions. Furthermore, we confirmed that the heavy metal composition proportions varied across measurement sites—the concentrations of Mn, Cu, Zn, As, Mo, Pb significantly differed between the two regions. The discrepancies in these concentrations may be attributed to the differences in the predominant industries of the industrial complexes in Dangjin and Yeosu·Gwangyang.

The health risk assessment found that PM_2.5_ exceeded the threshold of 1 at all measurement sites in both Dangjin and Yeosu·Gwangyang. Meanwhile, Mn exceeded the threshold of 1 at D1, D2, D3, D6, and Y3, indicating a potential health impact. Cd exceeded the threshold of 1.00 × 10^−6^ at D3, Y2, Y4, and Y5 in adult men and at D2, Y2, and Y5 in adult women, showing that the elements that exceed the threshold differ across measurement sites.

The findings of this study confirmed that PM_2.5_ compositions vary across regions and different measurement sites within the same region and, moreover, that health risks differ across regions due to these variations. These results highlight the need to consider PM_2.5_ concentrations as well as their compositions in PM_2.5_ exposure and health risk assessments. This study is significant in that it responds to the problem of not being able to conduct health risk assessment for PM_2.5_ due to a lack of PM_2.5_ toxicity data by using the Korean annual average atmospheric PM_2.5_ concentration as the toxicity reference value. Thus, the fiTablendings of this study can serve as foundational data for health risk assessments for PM_2.5_ and its components that consider the diverse characteristics of industrial complexes.

## Figures and Tables

**Table 1:** Specifications of analytical device.

Specifications for Analysis	
Angle between the X-ray tube and detector	80 °C
Distance between SDD detector and sample	≤15 mm
Distance between X-ray tube target and sample	≤40 mm
X-ray generator voltage range	4~50 kV
X-ray generator current range	0~3.0 mA
Resolution	140 eV@Mn Ka 100 kcps
Maximum	1.5 Mcps
Window	8 μm (0.315 mil) Beryllium

**Table 2:** Toxicity of PM_2.5_ and five heavy metals.

Pollutants	Non-Carcinogenic	Carcinogenic
	RfC ^1^ (mg/m^3^)	Inhalation Unit Risk ((μg/m^3^)^−1^)
PM_2.5_	0.015	-
Mn	5.00 × 10^−5^	-
Ni	-	2.40 × 10^−4^
As	-	4.30 × 10^−3^
Cd	-	1.80 × 10^−3^
Cr^6+^	1.00 × 10^−4^	1.20 × 10^−2^

^1^ Reference concentration.

**Table 3:** The exposure factor values used in this study.

	Unit	Value for Age Categories			Reference
		Children	Adult Men	Adult Women	
IR ^1^	m^3^/day	12.73	16.21	13.03	[19,20]
EF ^2^	day/year	365			This study
ED ^3^	year	6	24	24	This study
BW ^4^	kg	10.42	71.5	57.7	[19,20]
AT ^5^	day	2190	8760	8760	This study
LT ^6^	day	30,514	30,514	30,514	[21]

^1^ Inhalation rate. ^2^ Exposure frequency. ^3^ Exposure duration. ^4^ Body weight. ^5^ Average time. ^6^ Lifetime.

## Data Availability

Restrictions apply to the availability of these data. Data were obtained from MoE and are available with the permission of MoE.

## Supplementary Materials

The following supporting information can be downloaded at: https://www.mdpi.com/article/10.3390/toxics12020158/s1.
