# Supplementary material for: A Comparison of Health Risks from PM2.5 and Heavy Metal Exposure in Industrial Complexes in Dangjin and Yeosu·Gwangyang
Source: Toxics. 2024 Feb 18;12(2):158. doi: 10.3390/toxics12020158 (PMC10893162; doi:10.3390/toxics12020158)
Supplement: Supplementary file 1 [file toxics-12-00158-s001.zip › toxics-2843517-supplementary.pdf]

A Comparison of Health Risks from $PM_{2.5}$ and Heavy Metal Exposure in Industrial Complexes in Dangjin and Yeosu·Gwangyang

Jeong-In Jeon 1 , Ji-Yun Jung 1, Shin-Young Park 1 , Hye-Won Lee 2, Jeong-Il Lee 3 and Cheol-Min Lee 1,2,3,*

1 Department of Chemical and Environmental Engineering, Seokyeong University,

Seoul 02713, Republic of Korea; hhzz01@skuniv.ac.kr (J.-I.J.); jju1049@skuniv.ac.kr (J.-Y.J.);

tlsdud060900@skuniv.ac.kr (S.-Y.P.)

2 Institute of Environment and Health, Seoul 02713, Republic of Korea; gpdnsis@naver.com

3 Department of Nano, Chemical and Biological Engineering, Seokyeong University,

Seoul 02713, Republic of Korea; emjilee@skuniv.ac.kr

* Correspondence: cheolmin@skuniv.ac.kr

Table S1. Latitude and Longitude coordinates of measurement sites with sample sizes.

**Region**	**Sites**	**Coordinate**		**Number of samples**
		**Latitude**	**Longitude**	
Dangjin	D1	36.9418	126.7845	175
	D2	36.9854	126.7461	149
	D3	36.9575	126.6897	172
	D4	36.9821	126.5910	192
	D5	36.8932	126.6272	180
Yeosu· Gwangyang	Y1	34.9433	127.7496	193
	Y2	34.9374	127.6787	189
	Y3	34.8819	127.5787	194
	Y4	34.7983	127.6319	188
	Y5	34.8341	127.7350	191

Table S2. The results for quality assurance and quality control.

		**MDL [1]**	**RSD [2] (%)**
Heavy metals (ng/cm^2)	Al	59.28	0.59
	Ti	5.64	0.51
	V	17.14	1.27
	Mn	6.53	0.38
	Fe	8.08	0.59
	Ni	4.07	0.28
	Co	3.19	0.19
	Cu	3.43	0.35
	Zn	1.48	0.35
	As	1.37	0.31
	Sr	3.15	0.43
	Mo	4.29	0.43
	Cd	7.69	1.13
	Ba	3.86	0.12
	Pb	3.28	0.41
	P	1.84	0.26
	S	40.68	0.31
	Cr	1.12	0.10
	Si	6.20	0.39

[1] Method detection limit

[2] Relative standard deviation

Table S3. $PM_{2.5}$ (μg/m^3) and trace elemental concentration (ng/m^3) by measurement site.

	**Mean ± S.D. (Min-Max)**									
	**D1**	**D2**	**D3**	**D4**	**D5**	**Y1**	**Y2**	**Y3**	**Y4**	**Y5**
$PM_{2.5}$	24.8±13.7	24.4±14.2	21.8±12.4	20.9±12.7	21.4±13.1	16.7±8.7	16.2±10.1	19.0±9.8	17.2±9.0	18.1±9.6
	(4.7-76.1)	(4.1-73.6)	(4.8-76.9)	(3.9-73.5)	(4.7-80.0)	(3.1-60.5)	(3.2-94.5)	(3.6-64.6)	(4.8-59.4)	(3.4-68.4)
Al	403.9±669.2	356.1±659.8	389.5±619.2	361.1±550.1	353.8±534.1	298.3±387.3	370.3±547.8	353.0±547.8	373.2±563.6	380.7±539.0
	(4.2-3680.0)	(1.0-3629.8)	(23.4-3191.5)	(9.2-2746.2)	(0.6-2461.7)	(11.0-2413.0)	(19.5-2798.5)	(6.8-3422.4)	(12.4-3443.0)	(0.7-3391.7)
Ti	11.1±13.3	10.0±12.5	10.4±12.3	9.8±11.2	9.3±10.4	8.0±8.7	11.0±15.5	10.5±13.8	9.6±12.7	8.7±11.0
	(1.1-140.6)	(0.5-138.9)	(1.0-122.7)	(0.5-104.3)	(0.2-94.3)	(0.2-93.2)	(0.2-161.5)	(0.1-124.3)	(0.1-131.0)	(0.1-119.6)
V	1.6±1.2	2.0±1.9	1.4±1.1	1.4±1.1	1.1±0.8	1.6±1.3	1.4±1.0	1.2±0.8	1.3±0.9	1.6±1.1
	(0.1-6.5)	(0.2-10.4)	(0.1-6.4)	(0.1-6.2)	(0.1-4.1)	(0.1-7.7)	(0.1-5.8)	(0.1-1.1)	(0.1-5.2)	(0.1-5.4)
Mn	22.3±14.0	18.1±11.5	17.2±10.3	13.6±9.1	13.9±8.4	13.3±7.0	12.0±9.9	16.1±12.2	10.4±7.4	9.0±5.9
	(2.9-78.7)	(1.3-69.6)	(1.4-52.8)	(0.4-48.8)	(0.6-55.6)	(3.2-38.9)	(0.7-105.2)	(0.2-61.1)	(0.1-50.0)	(0.4-42.9)
Fe	255.7±177.9	223.2±162.2	165.6±130.6	144.5±120.0	134.7±110.6	268.3±147.7	172.6±149.8	178.1±137.8	159.9±134.6	127.5±117.6
	(28.9-1549.0)	(21.2-1436.4)	(19.1-1211.5)	(6.7-1063.4)	(9.4-912.3)	(32.3-1158.8)	(12.6-1267.7)	(9.2-1347.9)	(5.7-1371.8)	(5.6-1216.3)
Ni	1.9±1.4	2.2±1.7	1.5±1.0	1.7±1.2	1.5±1.5	2.5±2.4	1.5±1.1	1.7±1.1	1.6±1.1	1.7±1.4
	(0.1-9.4)	(0.2-13.1)	(0.1-6.3)	(0.1-7.0)	(0.1-13.9)	(0.1-19.5)	(0.1-7.0)	(0.1-7.2)	(0.1-9.1)	(0.1-14.5)
Co	1.5±1.0	1.3±0.9	1.0±0.8	1.7±1.2	1.5±1.5	2.5±2.4	1.5±1.1	1.7±1.1	1.6±1.1	1.7±1.4
	(0.1-8.2)	(0.1-7.7)	(0.1-6.3)	(0.1-7.0)	(0.1-13.9)	(0.1-19.5)	(0.1-7.0)	(0.1-7.2)	(0.1-9.1)	(0.1-14.5)
Cu	9.5±7.5	9.6±10.3	10.5±12.4	41.5±75.0	9.7±12.2	3.9±3.6	4.1±4.8	3.8±2.5	3.7±3.1	4.0±2.8
	(0.8-42.4)	(0.1-66.0)	(0.5-68.5)	(0.1-654.4)	(0.2-67.8)	(0.4-46.8)	(0.3-58.9)	(0.2-21.3)	(0.1-23.3)	(0.1-17.8)
Zn	83.0±43.6	68.9±47.9	58.2±37.0	56.3±45.5	54.7±40.1	54.3±29.1	39.8±26.2	47.5±32.8	41.4±27.3	35.6±25.3
	(8.5-234.1)	(4.0-364.9)	(3.5-184.3)	(0.8-279.3)	(1.7-210.9)	(11.1-185.1)	(2.4-162.8)	(0.9-200.6)	(1.5-153.0)	(0.9-162.7)
As	3.9±4.8	4.2±4.8	4.0±4.6	4.1±4.6	3.9±4.1	4.6±3.0	2.4±1.7	2.6±2.0	2.5±1.8	2.3±1.7
	(0.1-36.6)	(0.2-32.3)	(0.02-33.6)	(0.03-30.1)	(0.1-28.0)	(0.3-16.1)	(0.2-13.8)	(0.1-15.4)	(0.1-13.4)	(0.1-14.3)
Sr	1.4±1.1	1.3±1.1	1.4±1.3	1.5±1.2	1.3±1.1	1.2±1.0	1.7±4.5	1.3±1.1	1.2±1.0	1.3±1.0
	(0.1-6.3)	(0.02-6.8)	(0.02-6.1)	(0.1-6.9)	(0.02-5.7)	(0.1-4.3)	(0.1-46.6)	(0.03-6.1)	(0.1-6.1)	(0.1-5.5)
Mo	1.9±1.9	2.3±4.3	1.8±1.7	1.8±2.2	2.1±2.5	2.0±1.8	2.3±2.5	2.6±2.3	3.3±3.6	4.4±4.2
	(0.1-9.0)	(0.1-32.1)	(0.2-7.2)	(0.04-13.4)	(0.2-17.8)	(0.02-10.5)	(0.03-9.5)	(0.1-9.5)	(0.1-22.4)	(0.1-29.8)
Cd	3.4±3.8	3.6±3.4	4.1±4.0	3.8±4.5	3.7±4.0	3.8±4.0	4.5±4.6	3.6±3.7	3.6±3.6	3.5±3.3
	(0.1-22.1)	(0.1-14.1)	(0.1-17.1)	(0.02-22.5)	(0.02-18.0)	(0.03-19.9)	(0.02-19.1)	(0.1-16.9)	(0.02-18.1)	(0.1-15.6)
Ba	10.3±6.8	10.1±7.7	9.6±8.9	10.0±8.0	11.3±12.4	8.2±6.4	9.9±8.4	10.0±8.4	9.8±7.7	9.1±7.5
	(0.2-39.8)	(0.2-29.1)	(0.2-46.0)	(0.2-48.2)	(0.1-70.2)	(0.1-41.2)	(0.1-45.5)	(0.2-45.3)	(0.02-37.3)	(0.1-47.0)
P	10.1±9.0	9.6±9.1	8.2±8.1	8.4±8.1	8.5±7.6	8.03±6.7	9.2±10.5	8.1±8.6	7.9±9.4	13.7±3.7
	(0.2-54.8)	(0.1-54.8)	(0.1-50.6)	(0.03-46.4)	(0.1-43.4)	(0.3-41.6)	(0.3-73.6)	(0.2-61.2)	(0.03-72.8)	(0.1-84.4)
S	1197.2±929.9	1176.4±889.2	1080.9±814.0	1047.8±765.5	1052.3±818.5	1123.6±890.4	1090.0±881.2	1136.9±894.8	1068.2±826.1	1334.2±1001.1

	(102.2-4909.6)	(1109.1-4857.8)	(143.5-5239.3)	(123.8-5232.3)	(9.6-4897.8)	(2.5-7356.8)	(122.5-7470.8)	(85.4-7035.3)	(232.4-7556.9)	(7.1-8539.4)
Cr	3.0±2.3	3.0±2.0	2.4±1.4	1.5±0.03	2.3±2.2	3.3±2.8	2.2±1.6	2.5±1.6	2.4±1.7	1.9±1.3
	(0.3-22.9)	(0.2-16.3)	(0.2-8.5)	(0.03-7.6)	(0.1-22.3)	(0.3-16.0)	(0.2-10.5)	(0.2-8.0)	(0.1-12.0)	(0.1-6.6)
Si	400.1±633.8	377.6±601.6	360.2±570.4	343.5±505.9	339.6±494.1	311.1±424.4	402.1±745.9	333.9±519.4	333.6±538-8	381.0±529.0
	(2.4-6458.8)	(8.5-6312.7)	(1.1-5652.3)	(5.3-4901.8)	(10.5-4448.9)	(2.5-4513.1)	(0.8-8336.6)	(3.2-6093.9)	(9.8-6244.1)	(6.5-5947.8)

Table S4. Mean concentration of pollutants in Dangjin and Yeosu·Gwangyang.

	Unit	Area	Arithmetic mean	S.D.	p-value
$PM_{2.5}$	μg/m³	Dangjin	22.65	13.24	**p<0.05**
		Yeosu·Gwangyang	17.45	9.49	
Al	ng/m³	Dangjin	374.07	612.04	p>0.05
		Yeosu·Gwangyang	355.18	522.35	
Ti		Dangjin	9.94	11.96	p>0.05
		Yeosu·Gwangyang	9.19	12.47	
V		Dangjin	1.37	1.32	p>0.05
		Yeosu·Gwangyang	1.26	1.07	
Mn		Dangjin	16.88	11.28	**p<0.05**
		Yeosu·Gwangyang	12.14	9.12	
Fe		Dangjin	182.39	149.18	p>0.05
		Yeosu·Gwangyang	181.52	145.86	
Co		Dangjin	0.96	0.89	p>0.05
		Yeosu·Gwangyang	0.90	0.87	
Cu		Dangjin	16.77	38.77	**p<0.05**
		Yeosu·Gwangyang	3.89	3.45	
Zn		Dangjin	63.82	44.27	**p<0.05**
		Yeosu·Gwangyang	43.98	29.02	
As		Dangjin	4.02	4.58	**p<0.05**
		Yeosu·Gwangyang	2.71	2.32	
Sr		Dangjin	0.80	1.12	p>0.05
		Yeosu·Gwangyang	0.76	1.79	
Mo		Dangjin	1.94	2.56	**p<0.05**
		Yeosu·Gwangyang	3.02	3.22	
Cd		Dangjin	2.40	3.65	p>0.05
		Yeosu·Gwangyang	3.78	3.87	
Ba		Dangjin	6.67	8.70	p>0.05
		Yeosu·Gwangyang	5.76	7.60	
Pb		Dangjin	21.11	29.69	**p<0.05**
		Yeosu·Gwangyang	9.38	15.92	
P		Dangjin	6.50	8.19	p>0.05
		Yeosu·Gwangyang	6.84	9.74	
S		Dangjin	1106.29	848.85	p>0.05
		Yeosu·Gwangyang	1144.84	907.64	
Cr		Dangjin	2.55	1.96	p>0.05
		Yeosu·Gwangyang	2.41	1.94	
Si		Dangjin	360.47	560.88	p>0.05
		Yeosu·Gwangyang	344.15	557.84	

Table S5. Health risk assessment for non-carcinogens.

	Area	Site	HQ		
			Children	Adult-men	Adult-women
$PM_{2.5}$	Dangjin	D1	6.22E+00	1.15E+00	1.15E+00
		D2	6.07E+00	1.13E+00	1.12E+00
		D3	5.35E+00	9.92E-01	9.88E-01
		D4	4.91E+00	9.11E-01	9.08E-01
		D5	4.95E+00	9.19E-01	1.06E+00
	Yeosu·Gwangyang	Y1	4.18E+00	7.77E-01	7.74E-01
		Y2	3.96E+00	7.35E-01	7.32E-01
		Y3	4.76E+00	8.83E-01	8.80E-01
		Y4	4.43E+00	8.22E-01	8.19E-01
		Y5	4.52E+00	8.40E-01	8.36E-01
Mn	Dangjin	D1	1.60E+00	2.97E-01	2.96E-01
		D2	1.27E+00	2.35E-01	2.34E-01
		D3	1.20E+00	2.23E-01	2.22E-01
		D4	9.48E-01	1.76E-01	1.75E-01
		D5	1.06E+00	1.96E-01	1.96E-01
	Yeosu·Gwangyang	Y1	9.96E-01	1.85E-01	1.84E-01
		Y2	8.81E-01	1.63E-01	1.63E-01
		Y3	1.05E+00	1.96E-01	1.95E-01
		Y4	7.69E-01	1.43E-01	1.42E-01
		Y5	7.24E-01	1.34E-01	1.34E-01
Cr^{6+}	Dangjin	D1	9.44E-03	1.75E-03	1.75E-03
		D2	8.72E-03	1.62E-03	1.61E-03
		D3	6.96E-03	1.29E-03	1.29E-03
		D4	6.38E-03	1.18E-03	1.18E-03
		D5	6.05E-03	1.12E-03	1.12E-03
	Yeosu·Gwangyang	Y1	9.24E-03	1.71E-03	1.71E-03
		Y2	6.64E-03	1.23E-03	1.23E-03
		Y3	7.59E-03	1.41E-03	1.40E-03
		Y4	6.60E-03	1.23E-03	1.22E-03
		Y5	5.99E-03	1.11E-03	1.11E-03

Table S6. Health risk assessment for carcinogens.

	Area	Site	ECR		
			Children	Adult-men	Adult-women
Ni	Dangjin	D1	1.18E-07	8.75E-08	8.71E-08
		D2	1.32E-07	9.79E-08	9.75E-08
		D3	9.61E-08	7.13E-08	7.11E-08
		D4	1.06E-07	7.87E-08	7.84E-08
		D5	8.25E-08	6.12E-08	6.10E-08
	Yeosu·Gwangyang	Y1	1.47E-07	1.09E-07	1.08E-07
		Y2	9.21E-08	6.83E-08	6.81E-08
		Y3	1.08E-07	7.98E-08	7.95E-08
		Y4	1.07E-07	7.93E-08	7.90E-08
		Y5	1.03E-07	7.65E-08	7.62E-08
As	Dangjin	D1	3.19E-06	2.37E-06	2.36E-06
		D2	3.56E-06	2.64E-06	2.63E-06
		D3	3.35E-06	2.49E-06	2.48E-06
		D4	3.79E-06	2.81E-06	2.80E-06
		D5	3.40E-06	2.52E-06	2.51E-06
	Yeosu·Gwangyang	Y1	5.19E-06	3.85E-06	3.84E-06
		Y2	2.86E-06	2.13E-06	2.12E-06
		Y3	3.03E-06	2.25E-06	2.24E-06
		Y4	2.80E-06	2.08E-06	2.07E-06
		Y5	2.69E-06	2.00E-06	1.99E-06
Cd	Dangjin	D1	1.15E-06	8.53E-07	8.50E-07
		D2	1.35E-06	1.00E-06	1.00E-06
		D3	1.24E-06	9.18E-07	9.15E-07
		D4	1.10E-06	8.18E-07	8.15E-07
		D5	1.30E-06	9.66E-07	9.62E-07
	Yeosu·Gwangyang	Y1	1.32E-06	9.80E-07	9.76E-07
		Y2	1.32E-06	1.21E-06	1.20E-06
		Y3	1.25E-06	9.27E-07	9.23E-07
		Y4	1.35E-06	1.00E-06	9.97E-07
		Y5	1.43E-06	1.06E-06	1.06E-06
Cr^{6+}	Dangjin	D1	8.13E-07	6.04E-07	6.01E-07
		D2	7.51E-07	5.58E-07	5.55E-07
		D3	6.00E-07	4.45E-07	4.43E-07
		D4	5.49E-07	4.08E-07	4.06E-07
		D5	5.21E-07	3.87E-07	3.86E-07
	Yeosu·Gwangyang	Y1	7.95E-07	5.90E-07	5.88E-07
		Y2	5.72E-07	4.24E-07	4.23E-07
		Y3	6.54E-07	4.85E-07	4.84E-07
		Y4	5.69E-07	4.22E-07	4.20E-07
		Y5	5.16E-07	3.83E-07	3.81E-07
